# Pure Interstitial 7q21.3-q 31.1 Duplication: A Rare Segmental Genomic Aneuploidy: Case Report and Review of Cases with Distal and Similar Segment Involved

**DOI:** 10.1055/s-0041-1729546

**Published:** 2021-06-14

**Authors:** Alessandra Di Nora, Germana Lena, Andrea Giugno, Alessia Di Mari, Pierluigi Smilari, Carmelo Minardi, Piero Pavone

**Affiliations:** 1 Department of Clinical and Experimental Medicine, Postgraduate Training Program in Pediatrics, University of Catania, Catania, Italy; 2 Department of Radiology, Postgraduate Training Program in Radiology, University of Catania, Catania, Italy; 3 Department of Pediatric and Pediatric Neurology, University of Catania, Catania, Italy; 4 Department of Anaesthesia and Intensive Care, University Hospital “G. Rodolico” of Catania, Catania, Italy

**Keywords:** 7q21.3-q31.1, array CGH, developmental delay, facial features, duplication

## Abstract

In children with developmental delay (DD) and neurologic impairment, diagnosis can be challenging because of the wide spectrum of causes. Since the last decade, the use of array comparative genomic hybridization (CGH) offered a great contribution to get a diagnosis in complex phenotypes. The chromosome 7 is subject of interest in medical genetics because of its frequent association with chromosome aberrations, rearrangements, and deletions involving clinical manifestations. We hereby reported a 3-year-old male child patient with severe neuro-DD, craniofacial dysmorphisms, and pulmonary stenosis, whose array CGH analysis disclosed a duplication of 14.4 Mb on chromosome 7 (7q21.3-7q31.1). By reviewing the current literature to date, we first reported on neurologic and dysmorphic anomalies related to this rearrangement which was not previously reported.

## Introduction

Individuals with duplication of long arm (q) have been uncommonly reported. The isolated, “pure,” 7q form has been classically classified according to the chromosomal anomaly involving either the entire arm or the interstitial, proximal, and distal portions of the segment. 1 2 18 Novales et al 14 distinguished the affected individuals according to the chromosome 7q segment with these results: those presenting interstitial duplication 7q22 to 7q31 showed facial features consisting of frontal bossing, long eyelashes, narrow palpebral fissures, epicanthus, hypertelorism, small nose, long upper lip and ocular impairment in absence of skeletal anomalies, cleft palate, and early death; those with 7q31 to 7qter showed developmental delay (DD), facial features as large fontanelle, narrow palpebral fissures, hypertelorism, small nose, cleft palate, micrognathia, low-set and malformed ears, moreover, skeletal anomalies and early death were also recorded; duplication 7q32 to 7ter was characterized by DD, facial features presenting with small nose, low-set ears, in absence of micrognathia and cleft palate, skeletal anomalies, neurologic symptoms, and early death.

In this article, we reported a 3-year-old male child patient with pure partial duplication of the long arm of chromosome 7 extending for 14.4 Mb from 7q21.3 to q31.1. Clinical manifestations observed in the child are reported and compared with the clinical features found in children with the distal segment involved and then with those children who showed the interstitial chromosome 7q segment involved with the aim to individuate possible clinical signs specific for each of the segment affected.

## Case Presentation

This 3-year-old male child patient is the third child of healthy, unrelated Italian parents. The two older siblings, a 5-year-old sister and a 7-year-old brother, are healthy. The family history is negative for genetic disorders. At the time of gestation, the mother was 32 years old and the father was 35 years old. The mother denied to have suffering by infectious disease during her pregnancy and do not have smoked cigarettes or taken drugs during her pregnancy. Intrauterine ultrasound showed growth restriction by the 7th month and the mother noted that fetal movements were reduced. The child was delivered at 39 weeks of gestation by cesarean section due to breech presentation. His birth weight was 2,350 g (3th percentile), length 48 cm (10th percentile), and occipitofrontal circumference (OFC) 33 cm (3th percentile). At the second day of life, the child suffered by respiratory distress and he was admitted to Neonatal Intensive Care Unit, Department of the Pediatric Clinic, University of Catania, Italy for treatment and investigations. Cranial ultrasound displayed light hypodensity of the periventricular white matter. Echocardiogram showed pulmonary valve stenosis with atrial septal defect. At 10 days from birth, he was discharged in good condition without indication for a prompt cardiac intervention. During the first month, his clinical picture was mostly characterized by minor facial features and delayed developmental milestones particularly as regards hypotonia. At the age of 2.5 years, the child was admitted to the Pediatric Department of Catania University, Italy, for checkup due to DD and craniofacial anomalies. At this age, the weight was 12 kg (10th percentile), length 88 cm (10th percentile), and OFC 47 cm (3th percentile). According to the “Elements of Morphology: Standard Terminology” ( *Am J Med Genet* 2009, 149A (1): 1–127), he showed craniofacial features consisting of microcephaly, turricephaly, prominent frontal bossing, arched eyebrow shaved in the medial part, inner epicanthic folds, down-slanting palpebral fissure, flat nasal bridge, thin nose with rounded tip and anteverted nostrils, flat filter, thin lips, low implanted ears with rotated axis, and thin hair.

**Fig. 1:**
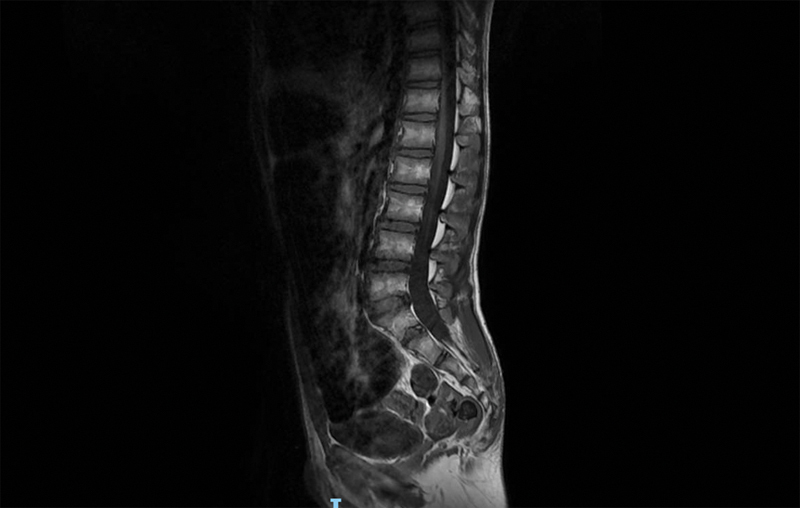
Spine magnetic resonance imaging showing S5 posterior arch cleft and distal sacral lipomatosis.

**Fig. 2:**
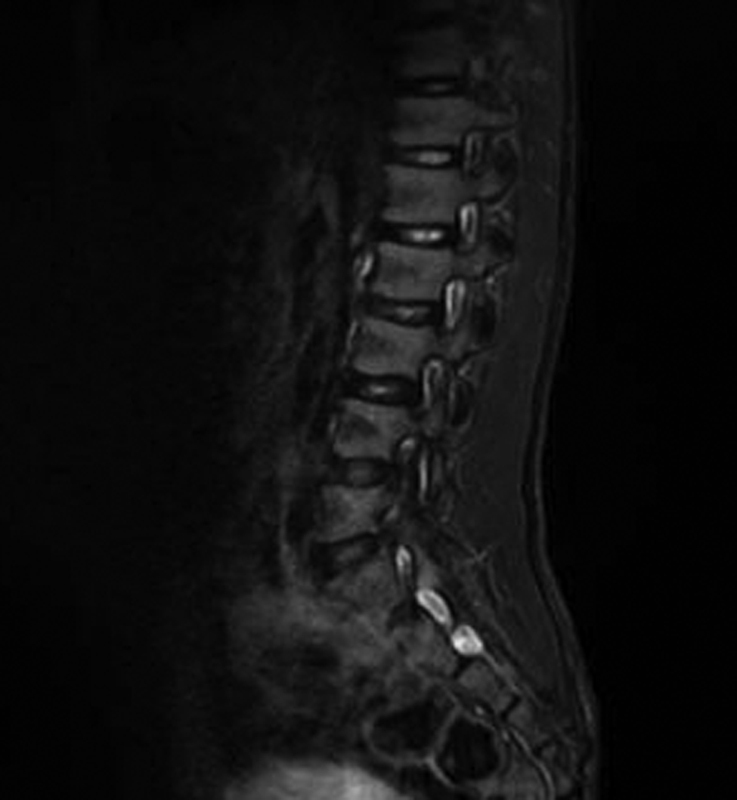
Spine magnetic resonance imaging showing S2 periapical cyst.

Muscular masses were hypotonic with poor subcutaneous tissue. He showed ankles valgism, knees in arthrogryposis, keeled chest, scapular winging, hands joint laxity, and abdominal rectus diastasis. DD was present, he was able to pronounce a very few words. The walk was unsteady. Patellar osteotendinous reflexes were hypo-eligible. Systolic murmur 2/6 Levine was appreciable and echocardiogram confirmed the presence of pulmonary valve stenosis with atrial septal defect. Left cryptorchidism was present at the routine laboratory analysis electrolytes, plasma and urinary amino acids, thyroid markers, organic acids, plasma purine, transferrin isoelectric focusing, and total cholesterol were within normal limits. Electroencephalogram in awake and during sleep was normal. Brain magnetic resonance imaging (MRI) with diffusion-weighted imaging sequences showed a regular volume and anatomy of the subtentorial and supratentorial structures and ventricles. The spine MRI pointed out a cleft in the S5 posterior arch, a distal sacral lipomatosis from S3 to S5, and a small periapical cyst Tarlov type at the right S2 ( Figs. 1 and 2 ).

## Genetic Testing

High definition karyotype analysis was performed after culture of lymphocytes for the child and his parents, according to the International System for Human Cytogenetic Nomenclature; 11 mean resolution was 550 bands; Cytocell painting probe was used for molecular cytogenetic.

Array comparative genomic hybridization (CGH) (human DNA, Promega) analysis using Cytofure ISCA 8 × 60K v.2 (protein O-GicNac Transferase, OGT) was performed on genetic DNA extracted from peripheral blood samples from the child and his parents. Software analysis was Cytofure Analysis Software (genomic assembly University of California, Santa Cruz, UCSC hg 19). Quality score (Derivative Log Ratio Spread, DLRS) was <0.25. Analysis parameter: four consecutive probes. Resolution: 50 to 100 Kb. Next-generation sequencing (NGS) panel for RASopathies was performed.

## Results

**Fig. 3:**
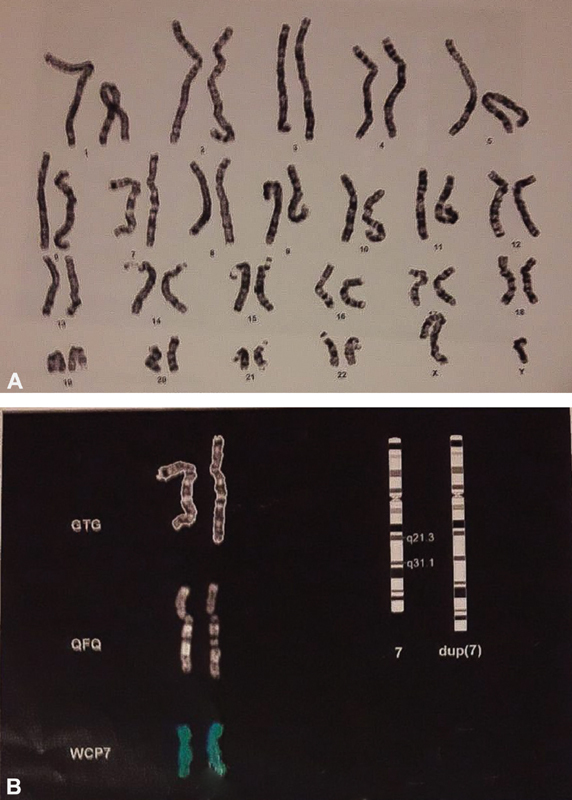
( **A,B** ) Karyotype of the child showing intrachromosomal partial duplication of the long arm of chromosome 7.

**Fig. 4:**
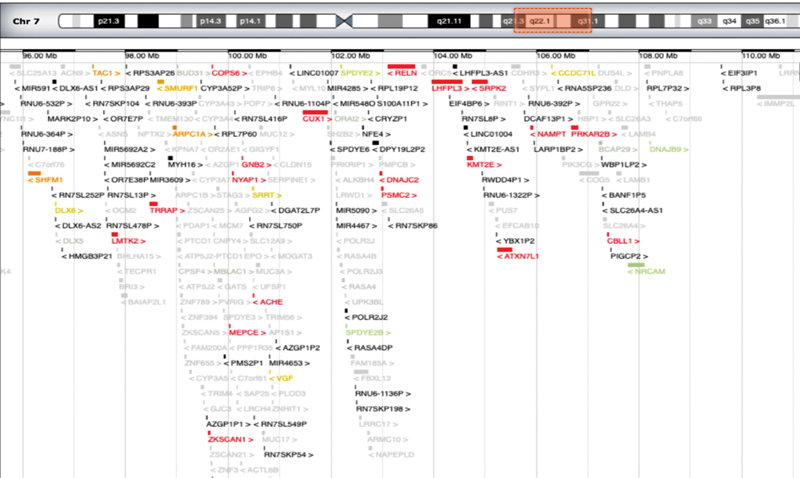
Imaging modified from OMIM showing genes included in the rearranged region.

Analyses showed in the child a 46, XY karyotype with intrachromosomal partial duplication of the long arm of chromosome 7, extending for 14.4 Mb (7q21.3-q31.1): arr [hg 18] ( Fig. 3A, B ). The centromeric margin of the duplication is made by the normal oligomer in position 95054968pb and the duplicated oligomer in position 109476740pb. The telomeric margin is made by the normal oligomer in position 109557905pb and the duplicated oligomer in 109476740bp ( Fig. 4 ). RASopathies NGS analysis did not show any alteration. Parents' karyotype and array CGH were normal.

## Discussion

**Fig. 5:**
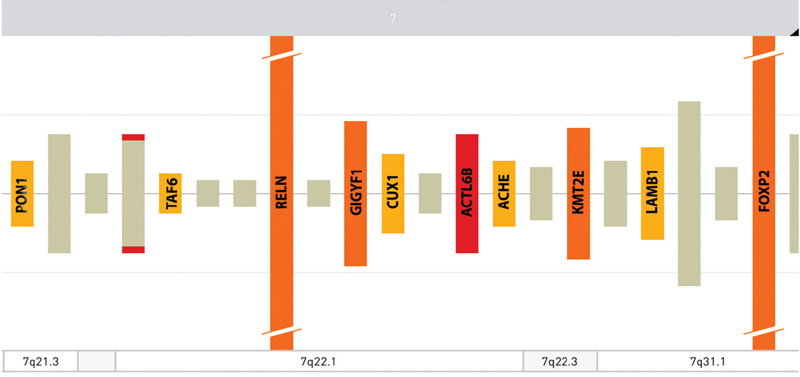
Imaging modified from SFARI genes showing genes involved in autism spectrum disorder high confidence.

Our patient showed a complex of congenital anomaly consisting of minor, nonspecific craniofacial features, microcephaly, mild DD, and heart and skeletal impairment. Genital organs, hands, and abdomen were also involved. Congenital heart disorder (CHD) consisted of pulmonary valve stenosis with septal defect; skeletal anomalies showed cleft of S5 posterior arch, distal sacral lipomatosis from S3 to S5, and right S2 periapical cyst Tarlov type; knees in arthrogryposis and ankles valgism were also present. In addition, abdominal rectus muscle diastasis, left cryptorchidism, and hands joint laxity were noted. Array CGH showed a large genomic duplication of 14.4 Mb on chromosome 7q21.q31.1 harboring several OMIM and RefSeq genes: the rearranged region includes more than 230 genes ( Fig. 4 ). According to SFARI gene database, some of those such as *ACTL6B* , *FOXP2* , *PONI* , *KMT2E* are involved in synaptic plasticity and autistic signaling mechanisms and other structural anomalies ( Fig. 5 ) with causative effect in congenital disorders affecting brain and other organs.

Chromosome 7q is susceptible to different structural rearrangements. Attempt to highlight specific clinical signs according to each of the interstitial, proximal, or distal segment involved has been not clearly defined. Alfonsi et al tried to compare the clinical features of a case of their observation presenting partial chromosome 7q duplication involving the region q21.1-q22.3 with six patients extracted from the literature with similar 7q rearrangement. 1 These authors found concomitant presence of intellectual delay and low-set malformed ears in all the cases including their own; ocular squint and frontal bossing features in six; and skeletal anomalies in five. Three of the patients showed genital urinary defects, macrocephaly, and some similar craniofacial features such as hypertelorism, small upturned nose, microretrognathia, cleft palate, and high arched palate. The authors draw the conclusion that in individuals with pure chromosome 7q proximal segment involved, specific diagnostic clinical features were not raised. 1

In this study, we analyzed clinical data of each of the eight cases of 7q pure duplication interstitial (7q22 to 7q32) segment reported in the literature including the present case to evaluate clinical similarity or differences ( Table 1 ). In the interstitial groups, no gender prevalence was noted as three males and four females were the children affected. DD and minor, nonspecific facial features were reported in all the cases. In particular, looking at some facial features, frontal bossing and epicanthus were reported in six out of seven, low-set ears in four, and microretrognathia in two cases. Hypotonia, growth retardation was found in six and ocular squint in five out of seven cases. Three children were microcephalic, and heart, skeletal, and genital anomalies were reported only in the present case but not in the others. Hirsutism and hip subluxation were reported in two cases and cerebral malformation and renal dysplasia only in one case. 2 4 7 15 17 18 21 Analyzing these results, it appears evident that except for intellectual delay and minor craniofacial delay, no specific clinical signs have been raised to indicate a clear diagnosis.

**Table 1:** Clinical data of eight cases presenting pure 7q distal duplication 1 3 6 7 8 9 10 in comparison with the present case presenting pure 7q interstitial duplication

	Distal 7q	Present case
Gender	M/F: 7/1	M
Facial features	8/8	+
Large head	5/8	−
Developmental delay	8/8	+
ASD	1/8	−
Skeletal anomalies	4/8	+
Genitourinary anomalies	2/8	+
CHD	1/8	+

Abbreviations: ASD, autism spectrum disorder; CHD, congenital heart disorder.

We extended our study examining clinical data of pure distal versus pure interstitial 7q duplication comparing the six cases reported in the literature with those observed in the present child ( Table 2 ). 3 6 10 17 20 22 Analyzing these results, it appears evident that minor nonspecific craniofacial features, intellectual delay, and skeletal impairment with minor frequency were the most representative clinical signs presented both in the group of children with distal and in the present child with interstitial chromosome 7q involvement.

**Table 2:** Clinical data of the six reported cases (8, 11–15) plus the present case with pure interstitial 7q duplication

	Grace et al (1972)	Berger et al (1974)	Serville et al (1975)	Romain et al (1990)	Mégarbané et al (2000)	Weimer et al (2011)	Present case	Total
Chromosome 7	q22-q32	q21-q31	q22-q31	q22-q31.2	q22-q31.3	q21.1-q31.3	q21.3-q31.1	
Sex	F	M	M	F	F	F	M	3 M/4 F
Facial features								
Frontal bossing	−	+	+	+	+	+	+	6/7
Epicanthus	+	+	+	+	+	+	+	6/7
Microretrognathia	−	−	−	−	+	−	+	2/7
Low-set ears	+	−	+	−	+	−	+	4/7
Development delay	+	+	+	+	+	+	+	7/7
Microcephaly	−	−	−	−	+	+	+	3/7
Hypotonia	−	+	+	+	+	+	+	6/7
Growth retardation	+	−	+	+	+	+	+	6/7
CHD	−	−	−	−	−	−	+	1/7
Genital anomalies	−	−	−	−	−	−	+	1/7
Skeletal anomalies	−	−	−	−	−	−	+	1/7
Ocular anomalies	−	+	+	+	+	+	−	5/7
Other anomalies	−	−	+	+	+	+	+	5/7

Abbreviations: CHD, congenital heart disease; F, female; M, male.

Note: The other anomalies include: hirsutism reported in two cases (14 and 15); hearing loss and renal dysplasia in one; 8 and spine anomalies in the present case.

Aside pure, isolated chromosome 7q duplication, the 7q anomaly may occur in association with rearrangements involving the same or other chromosomes causing different and more complex clinical manifestations. Frühmesser et al reported a case of partial trisomy 7q22q32 with additional inversion of 7q31.2.2q32.3. 15 The child showed DD, growth failure, hands anomalies, and CHD presenting with patent ductus arteriosus and patent foramen ovale.

Craniofacial features consisted of frontal bossing, slightly down-slanting palpebral fissures, broad nasal root, long philtrum micrognathia, and prominent ears. Paththinige et al reported on a female child trisomic for 7q22.-qter and minimal loss of genetic material on chromosome 14. 13 Intrauterine growth retardation, DD, and ventriculomegaly were the main signs with craniofacial features presenting with asymmetric skull, triangular face shape, high forehead, hypertelorism, flat nasal bridge, micrognathia, and low-set malformed ears. 16 The authors agreed with the results of the literature that in this case, no clear correlation genotype–phenotype was found.

The present child showed a CHD consisting of a stenosis of the pulmonary valve, an anomaly which is reported as one of the typical signs of Noonan's syndrome. This syndrome is related to alterations involving the RAS-MAPK pathway 17 as it falls into a group of disorders called “RASopathies” which share common dysregulation of RAS/MAPK pathway. Due to some common clinical signs with the present child including the cardiac defect, we extended the genetic research with NGS panel for RASopathy principal genes which in our case gave negative results. In addition, the present child showed anomalies affecting the skeletal system, in particular, the spine with S5 posterior arch cleft, sacral lipomatosis, and sacrococcygeal anomalies. The anomalies (the last one) are part of the Currarino syndrome (CS).

CS known as autosomal dominant sacral agenesis is a well-known disorder characterized by a classic triad of partial absence of the sacrum with intact first sacral vertebra, presacral mass, and anorectal anomalies. 18 CS may manifest with a variable clinical expression ranging from only caudal anomalies to complex organ system malformation. The disorder has been related to a deleterious variants of *HLXB9* , a homeobox gene mapped to chromosome 7q36. 8 Pavone et al reported a female child with pre- and postnatal growth retardation and complex malformations with microcephaly, lack of cortical thickening, hypoplastic inferior vermis in association with signs of CS as partial sacral agenesis and complete coccygeal agenesis, preintrasacral dermoid, intradural lipoma, ectopic anus, and tethered cord. 19 20 21 22 The child showed a de novo duplication of 7q34-q35 and an 8.8-Mb deletion on 7q36. This sequence includes *HLXB9* (CS) gene which in the case report of Pavone et al was not found. 14 Clinical relationship between the spine anomalies reported in the present child and those reported in CS remain to be established.

## Conclusion

In conclusion, clinical signs and body organs presenting in individuals with involvement of the different proximal, interstitial, and distal pure segments of q7 duplication are nonspecific and not diagnostically indicative. The same results were reached for what it regards the single case with involvement the interstitial segment involved as with the exception of no distinctive facial features and mild DD nonspecific, indicative diagnostic signs were reported.
